# Risk factors, prognostic factors, and nomograms for distant metastases in patients with gastroenteropancreatic neuroendocrine tumors: a population-based study

**DOI:** 10.3389/fendo.2024.1264952

**Published:** 2024-02-14

**Authors:** Xinwei Li, Yongfei Fan, Jichun Tong, Ming Lou

**Affiliations:** ^1^ Department of Gastroenterology, Affiliated Cancer Hospital of Bengbu Medical College, Bengbu, China; ^2^ Department of Thoracic Surgery, The Affiliated Changzhou Second People’s Hospital of Nanjing Medical University, Changzhou, China

**Keywords:** gastroenteropancreatic neuroendocrine tumors, distant metastasis, nomogram, prognosis, SEER

## Abstract

**Background:**

Patients with gastroenteropancreatic neuroendocrine tumors (GEP-NETs) have a poor prognosis for distant metastasis. Currently, there are no studies on predictive models for the risk of distant metastasis in GEP-NETs.

**Methods:**

In this study, risk factors associated with metastasis in patients with GEP-NETs in the Surveillance, Epidemiology, and End Results (SEER) database were analyzed by univariate and multivariate logistic regression, and a nomogram model for metastasis risk prediction was constructed. Prognostic factors associated with distant metastasis in patients with GEP-NETs were analyzed by univariate and multivariate Cox, and a nomogram model for prognostic prediction was constructed. Finally, the performance of the nomogram model predictions is validated by internal validation set and external validation set.

**Results:**

A total of 9145 patients with GEP-NETs were enrolled in this study. Univariate and multivariate logistic analysis demonstrated that T stage, N stage, tumor size, primary site, and histologic types independent risk factors associated with distant metastasis in GEP-NETs patients (p value < 0.05). Univariate and multivariate Cox analyses demonstrated that age, histologic type, tumor size, N stage, and primary site surgery were independent factors associated with the prognosis of patients with GEP-NETs (p value < 0.05). The nomogram model constructed based on metastasis risk factors and prognostic factors can predict the occurrence of metastasis and patient prognosis of GEP-NETs very effectively in the internal training and validation sets as well as in the external validation set.

**Conclusion:**

In conclusion, we constructed a new distant metastasis risk nomogram model and a new prognostic nomogram model for GEP-NETs patients, which provides a decision-making reference for individualized treatment of clinical patients.

## 1 Introduction

Neuroendocrine neoplasms (NENs) are a heterogeneous group of tumors originating from neuroendocrine cells (1). The 2022 WHO classification categorizes these neoplasms, based on morphology and proliferation index, into well-differentiated neuroendocrine tumors (NETs), poorly differentiated neuroendocrine carcinomas (NECs), and mixed neuroendocrine-non-neuroendocrine neoplasms (MiNENs) (2–4). Within this classification, NETs are subdivided into Grade 1, Grade 2, and Grade 3, based on mitotic count and Ki-67 proliferation index, key indicators of tumor behavior and prognosis. It is important to note that the majority of extrapulmonary NENs are well-differentiated (NETs), while a smaller proportion, approximately 10-20%, are poorly differentiated (NECs) (2). Functioning NENs, which represent about 20% of these extrapulmonary neoplasms, are characterized by their hormone-secreting ability and the clinical symptoms resulting from hormone hypersecretion (1). In contrast, non-functioning NENs, which do not produce active hormones or cause related symptoms, account for the majority (80%) of extrapulmonary NENs (1). These neoplasms are most commonly found in the digestive system, including the stomach, intestines, and pancreas, representing about 60-70% of extrapulmonary NEN cases (1, 4–6). The prevalence and diversity of extrapulmonary NENs highlight the critical need for ongoing research and a nuanced understanding of their classification and behavior for effective management and treatment.

Gastroenteropancreatic NETs (GEP-NETs) have the second highest incidence of cancers of the digestive system (1, 7). In recent years, several studies have suggested a gradual increase of the incidence of GEP-NETs, with a six-fold increase in the incidence of GEP-NETs from 1997 to 2012 (6–9). GEP-NETs are a group of relatively slow-growing tumors (1). However, according to statistics, about 27% of GEP-NETs metastasize at the time of diagnosis (10). Due to the heterogeneity of GEP-NETs, tumor cell invasiveness varies across primary sites. NETs in the gastric and rectal sites have a low cellular metastatic capacity, but once metastasis occurs, the disease progresses rapidly, whereas NETs in the small intestinal site have a high malignant potential, but progress slowly after metastasis (1, 11–13). Studies have demonstrated that the median survival of localized NETs is more than 30 years, while distant metastases are only 12 months (10, 14). Currently, imaging is the primary modality for the diagnosis and staging of GEP-NETs. While it is crucial in assessing tumor spread, certain limitations exist in detecting distant metastases in GEP-NETs patients (4, 9, 15). Computed tomography (CT) has a detection rate of only 61% (46%-80%) for bone metastases, 79% (73%e94%) for liver metastases, and small peritoneal metastases are difficult to detect (4, 16). Magnetic resonance imaging (MRI) is superior to CT in detecting the liver, pancreas, bones, and brain, but it can also miss small metastases in the lungs (4, 15).

In this study, risk factors and prognostic factors associated with distant metastasis in patients with GEP-NETs were analyzed based on the Surveillance, Epidemiology, and End Results (SEER) database, a multicenter registry in the United States. Clinical diagnostic and prognostic models were established based on the risk factors and prognostic factors obtained from the analysis so as to guide clinical treatment and improve patient prognosis.

## 2 Materials and methods

### 2.1 Patients

**Figure 1:**
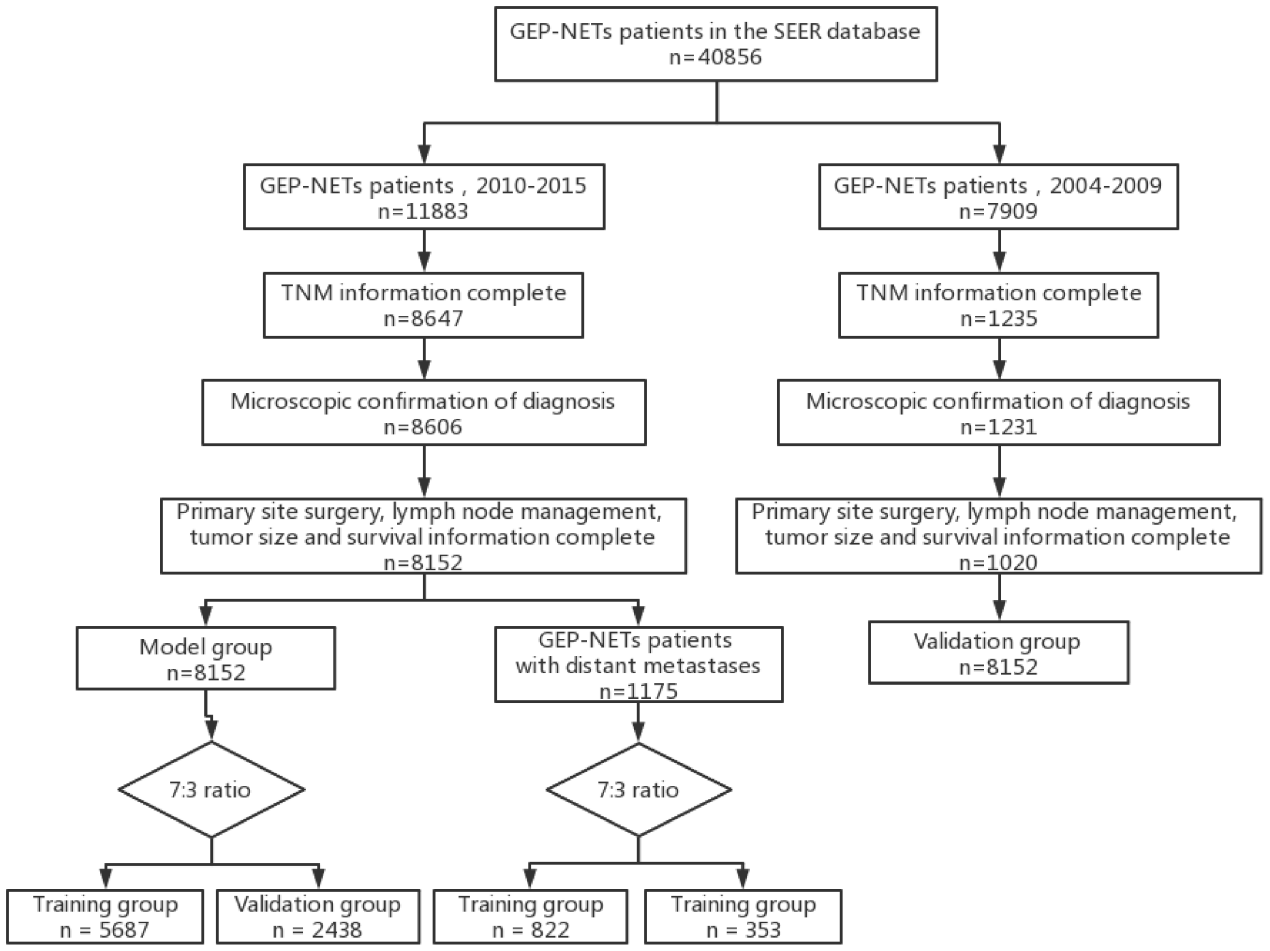
Flow chart depicting the patient selection process. GEP-NETs, gastroenteropancreatic neuroendocrine tumors; SEER, Surveillance, Epidemiology, and End Results; TNM, Tumor Node Metastasis.

In this study, clinical information of patients was obtained from the SEER database (https://seer.cancer.gov; accessed date July 1, 2023). In order to include more patient sample information to construct a more accurate model, taking into account the prognostic model 3-year survival index (2000-2017) and different American Joint Committee on Cancer (AJCC) version staging (6th edition: 2004-2015; 7th edition: 2010-2015; 8th edition: from 2018), we finally selected the 6th edition of AJCC staging for the present study. Patients diagnosed with GEP-NETs from 2010-2015 in the “Incidence - SEER Research Data, 17 Registries, Nov 2022 Sub (2000-2020)” database were selected as the risk and prognosis model construction group (model group), and patients diagnosed with GEP-NETs from 2004-2009 were selected as the model external validation group (validation group). Patients with GEP-NETs obtained from both databases were subjected to the same inclusion criteria as follows: (1) Patients with primary site International Classification of Diseases for Oncology, 3rd Edition (ICD-O-3): C16.0-C16.6; C16.8-C16.9; C17.0-C17.3; C17.8-C17.9; C18.0-C18.9; C19.9; C20.9; C21.0-C21.8; C25.0-C25.4; C25.7-C25. and pathology type ICD-O-3: 8150-8156; 8240-8244; 8246; 8249 in the database were selected. (2) Clinical information of patients included in the analysis included age, gender, year of diagnosis, race, histologic type, primary site, tumor size, T stage, N stage, M stage, primary site surgery, lymph node disposition, survival status, and follow-up time. (3) Exclusion of patients with GEP-NETs not diagnosed by microscopy. (4) To ensure the integrity of the study data, patients with unknowns in T stage, N stage, M stage, tumor size, primary site surgery, lymph node disposition, and follow-up time were removed (due to database limitations chemotherapy and radiotherapy data that included too much unknown information were excluded from this study). (5) To exclude non-tumor-related deaths, samples of patients with survival time less than 1 month were removed (**Figure 1**).

### 2.2 Constructing risk and prognosis related models and validation

Patients with GEP-NETs in the model group were randomly divided into training and validation sets in a ratio of 7:3. Risk factors associated with patients with distant metastases of GEP-NETs were analyzed by univariate and multivariate logistic regression. In the training set, a nomogram model was constructed for predicting the risk of metastasis in patients with GEP-NETs, and the accuracy and utility of the model were assessed using Receiver Operating Characteristic Curve (ROC) curves, calibration curves, and Decision Curve Analysis (DCA) curves. The validation set was used to verify the accuracy of the risk model constructed from the training set. In addition, external validation of the risk model was performed using validation group.

Patients with distant metastases of GEP-NETs in the model group were screened and divided into training and validation sets in a ratio of 7:3. Univariate and multivariate Cox analyses were used to find prognostic factors and develop prognostic-related nomogram prognostic models. First, the relationship between each factor and prognosis was assessed by univariate Cox analysis, and prognostically significant influences were screened based on a p value <0.05. The Hazard Ratio (HR) of each variable was calculated to quantify the prognostic impact of each factor. Then, the significant variables screened in the univariate Cox analysis were included in the multivariate Cox analysis to assess the independent effect of each variable on prognosis after controlling for other variables. Finally, the prognostic model was validated using an internal validation set and an external validation group. In addition, fitting clinical information with less sample size, such as histologic type and N stage, to construct a model with a more concentrated distribution of model parameters.

### 2.3 Statistical analysis

In this study, all statistical analyses were performed through R software (version 4.3.1.). The chi-square test was used to compare the distribution of clinical variables in the training and test sets. Differential survival of patients in high- and low-risk groups classified by prognostic model was compared by log-rank test. A p value < 0.05 was considered statistically significant.

### 2.4 Ethical statement

The SEER database is a public open database that does not include identifiable patient information. All patients were informed and signed written informed consent at the time of inclusion in the database and passed the ethical review of the local institution. In addition, this study was also approved by the Ethics Committee of Changzhou Second People’s Hospital affiliated with Nanjing Medical University.

## 3 Results

### 3.1 Baseline characteristics of the study population

A total of 9145 patients with GEP-NETs were enrolled in this study, including 8125 patients in the model group and 1020 patients in the validation group. The mean overall survival (OS) was 75.9 months (range from 1 to 131 months) in the model group and 85.3 months (range from 1 to 203 months) in the validation group. Both groups of patients had intestines (70.9% and 51.3%) and (22.1% and 41.7%) pancreas as the most common primary sites, with the neuroendocrine tumor (65.1%) histologic type being the most common in the model group and the neuroendocrine carcinoma (95.2%) histologic type being the most common in the validation group (**Table 1**).

**Table 1:** Clinical information distribution of GEP-NETs patients in the model and validation groups.

Variables	Model group	Validation group
	(n = 8125)	(n = 1020)
Status, (n, %)		
Alive	6099 (75.1%)	369 (36.2%)
Dead	2026 (24.9%)	651 (63.8%)
Survival, months (n, %)		
Mean	75.9	85.3
Median	79.0 [1.00, 131]	80.0 [1.00, 203]
Primary site surgery (n, %)		
No	949 (11.7%)	219 (21.5%)
Yes	7176 (88.3%)	801 (78.5%)
Lymph node dissection (n, %)		
No	4484 (55.2%)	362 (35.5%)
Yes	3641 (44.8%)	658 (64.5%)
T stage (n, %)		
T1	3946 (48.6%)	183 (17.9%)
T2	1523 (18.7%)	245 (24.0%)
T3	1864 (22.9%)	417 (40.9%)
T4	792 (9.7%)	175 (17.2%)
N stage (n, %)		
N0	5726 (70.5%)	478 (46.9%)
N1	2352 (28.9%)	424 (41.6%)
N2	46 (0.6%)	116 (11.4%)
N3	1 (0.0%)	2 (0.2%)
M stage (n, %)		
M0	6950 (85.5%)	646 (63.3%)
M1	1175 (14.5%)	374 (36.7%)
Tumor size, cm (n, %)		
< 2	5144 (63.3%)	255 (25.0%)
2 ~ 5	2209 (27.2%)	452 (44.3%)
>= 5	772 (9.5%)	313 (30.7%)
Primary site (n, %)		
Intestine	5764 (70.9%)	523 (51.3%)
Pancreas	1796 (22.1%)	425 (41.7%)
Stomach	565 (7.0%)	72 (7.1%)
Histologic type (n, %)		
Neuroendocrine tumor	5290 (65.1%)	0
Neuroendocrine carcinoma	2167 (26.7%)	971 (95.2%)
Other	668 (8.2%)	49 (4.8%)
Race (n, %)		
Black	1044 (12.8%)	103 (10.1%)
Other	1124 (13.8%)	103 (10.1%)
White	5957 (73.3%)	814 (79.8%)
Sex (n, %)		
Female	4036 (49.7%)	474 (46.5%)
Male	4089 (50.3%)	546 (53.5%)
Age, years (n, %)		
< 30	333 (4.1%)	12 (1.2%)
30 ~ 60	3848 (47.4%)	468 (45.9%)
>= 60	3944 (48.5%)	540 (52.9%)

GEP-NETs, gastroenteropancreatic neuroendocrine tumors.

Patients with GEP-NETs in the model group were divided into training (n = 5687) and test (n = 2438) sets according to 7:3, to exploring risk factors for distant metastasis of GEP-NETs, and to construct a prediction model for the risk of distant metastasis. Patients with distant metastases in the model group were divided into training (n = 822) and test (n = 353) sets according to 7:3, to exploring the prognostic factors of distant metastases of GEP-NETs, and to construct prognostic prediction model. The chi-square test indicated that this allocation was randomized (**Tables 2**, **3**; p value > 0.05).

**Table 2:** Clinical information distribution of the GEP-NETs patients in model group divided into training and test sets.

Variables	Overall	Training group	Validation group	Comparison
	(n = 8125)	(n = 5687)	(n = 2438)	
T stage (n, %)				
T1	3946 (48.6%)	2775 (48.8%)	1171 (48.0%)	χ ^2^ = 0.495 p = 0.920
T2	1523 (18.7%)	1065 (18.7%)	458 (18.8%)	
T3	1864 (22.9%)	1298 (22.8%)	566 (23.2%)	
T4	792 (9.7%)	549 (9.7%)	243 (10.0%)	
N stage (n, %)				
N0	5726 (70.5%)	4012 (70.5%)	1714 (70.3%)	χ ^2^ = 2.373 p = 0.499
N1	2352 (28.9%)	1643 (28.9%)	709 (29.1%)	
N2	46 (0.6%)	32 (0.6%)	14 (0.6%)	
N3	1 (0.0%)	0 (0%)	1 (0.0%)	
M stage (n, %)				
M0	6950 (85.5%)	4845 (85.2%)	2105 (86.3%)	χ ^2^ = 1.723 p = 0.189
M1	1175 (14.5%)	842 (14.8%)	333 (13.7%)	
Tumor size, cm (n, %)				
< 2	5144 (63.3%)	3632 (63.9%)	1512 (62.0%)	χ ^2^ = 2.518 p = 0.284
2 ~ 5	2209 (27.2%)	1524 (26.8%)	685 (28.1%)	
>= 5	772 (9.5%)	531 (9.3%)	241 (9.9%)	
Primary site (n, %)				
Intestine	5764 (70.9%)	4031 (70.9%)	1733 (71.1%)	χ ^2^ = 1.553 p = 0.460
Pancreas	1796 (22.1%)	1248 (21.9%)	548 (22.5%)	
Stomach	565 (7.0%)	408 (7.2%)	157 (6.4%)	
Histologic type (n, %)				
Neuroendocrine tumor	5290 (65.1%)	3692 (64.9%)	1598 (65.5%)	χ ^2^ = 0.741 p = 0.690
Neuroendocrine carcinoma	2167 (26.7%)	1518 (26.7%)	649 (26.6%)	
Other	668 (8.2%)	477 (8.4%)	191 (7.8%)	
Race (n, %)				
Black	1044 (12.8%)	741 (13.0%)	303 (12.4%)	χ ^2^ = 3.192 p = 0.203
other	1124 (13.8%)	808 (14.2%)	316 (13.0%)	
White	5957 (73.3%)	4138 (72.8%)	1819 (74.6%)	
Sex (n, %)				
Female	4036 (49.7%)	2850 (50.1%)	1186 (48.6%)	χ ^2^ = 1.413 p = 0.235
Male	4089 (50.3%)	2837 (49.9%)	1252 (51.4%)	
Age, years (n, %)				
< 30	333 (4.1%)	228 (4.0%)	105 (4.3%)	χ ^2^ = 1.808 p = 0.405
30 ~ 60	3848 (47.4%)	2720 (47.8%)	1128 (46.3%)	
>= 60	3944 (48.5%)	2739 (48.2%)	1205 (49.4%)	

GEP-NETs, gastroenteropancreatic neuroendocrine tumors.

**Table 3:** Clinical information distribution of GEP-NETs patients with distant metastases in the model group divided into training and test sets.

Variables	Overall	Training group	Validation group	Comparison
	(n =1175)	(n = 822)	(n = 353)	
Primary site surgery (n, %)				
No	409 (34.8%)	284 (34.5%)	125 (35.4%)	χ ^2^ = 0.047 p = 0.828
Yes	766 (65.2%)	538 (65.5%)	228 (64.6%)	
Lymph node dissection (n, %)				
No	483 (41.1%)	335 (40.8%)	148 (41.9%)	χ ^2^ = 0.096 p = 0.757
Yes	692 (58.9%)	487 (59.2%)	205 (58.1%)	
T stage (n, %)				
T1	53 (4.5%)	35 (4.3%)	18 (5.1%)	χ ^2^ = 1.720 p = 0.632
T2	280 (23.8%)	195 (23.7%)	85 (24.1%)	
T3	487 (41.4%)	335 (40.8%)	152 (43.1%)	
T4	355 (30.2%)	257 (31.3%)	98 (27.8%)	
N stage (n, %)				
N0	404 (34.4%)	275 (33.5%)	129 (36.5%)	χ ^2^ = 1.151 p = 0.562
N1	746 (63.5%)	530 (64.5%)	216 (61.2%)	
N2	25 (2.1%)	17 (2.1%)	8 (2.3%)	
Tumor size, cm (n, %)				
< 2	228 (19.4%)	157 (19.1%)	71 (20.1%)	χ ^2^ = 1.286 p = 0.526
2 ~ 5	615 (52.3%)	439 (53.4%)	176 (49.9%)	
>= 5	332 (28.3%)	226 (27.5%)	106 (30.0%)	
Primary site (n, %)				
Intestine	708 (60.3%)	502 (61.1%)	206 (58.4%)	χ ^2^ = 0.769 p = 0.681
Pancreas	436 (37.1%)	299 (36.4%)	137 (38.8%)	
Stomach	31 (2.6%)	21 (2.6%)	10 (2.8%)	
Histologic type (n, %)				
Neuroendocrine tumor	421 (35.8%)	290 (35.3%)	131 (37.1%)	χ ^2^ = 1.907 p = 0.385
Neuroendocrine carcinoma	631 (53.7%)	451 (54.9%)	180 (51.0%)	
Other	123 (10.5%)	81 (9.9%)	42 (11.9%)	
Race (n, %)				
Black	123 (10.5%)	84 (10.2%)	39 (11.0%)	χ ^2^ = 0.487 p = 0.784
other	95 (8.1%)	69 (8.4%)	26 (7.4%)	
White	957 (81.4%)	669 (81.4%)	288 (81.6%)	
Sex (n, %)				
Female	569 (48.4%)	406 (49.4%)	163 (46.2%)	χ ^2^ = 0.898 p = 0.343
Male	606 (51.6%)	416 (50.6%)	190 (53.8%)	
Age, years (n, %)				
< 30	13 (1.1%)	10 (1.2%)	3 (0.8%)	χ ^2^ = 0.658 p = 0.720
30 ~ 60	510 (43.4%)	361 (43.9%)	149 (42.2%)	
>= 60	652 (55.5%)	451 (54.9%)	201 (56.9%)	

GEP-NETs, gastroenteropancreatic neuroendocrine tumors.

### 3.2 Analysis of risk factors for distant metastasis in GEP-NETs patients

To explore the risk factors associated with distant metastasis in GEP-NETs patients, we included eight clinical variables for univariate and multivariate logistic analyses. Univariate analysis revealed that T stage, N stage, tumor size, primary site, histologic type, race, and age were risk factors associated with metastasis of GEP-NETs (**Table 4**; p value < 0.05). Multivariate analysis demonstrated that high T stage, high N stage, large tumor size, pancreatic primary site, and other histologic types (pathological subtypes of GEP-NETs besides neuroendocrine tumor and neuroendocrine carcinoma) were independent risk factors for distant metastasis of GEP-NETs (**Table 4**; odd ratio (OR) > 1; p value < 0.05).

**Table 4:** Univariate and multivariate logistic regression analysis of risk factors associated with distant metastasis in patients with GEP-NETs.

Variables	Univariate analysis			Multivariate analysis		
	HR	95% CI	p value	HR	95% CI	p value
T stage						
T1	Reference			Reference		
T2	16.546	12.939 - 21.440	p < 0.001	5.764	4.438 - 7.715	p < 0.001
T3	25.978	20.506 - 33.391	p < 0.001	7.980	6.005 - 10.705	p < 0.001
T4	59.670	46.482 - 77.617	p < 0.001	18.766	13.865 - 25.619	p < 0.001
N stage						
N0	Reference			Reference		
N1	6.119	5.474 - 6.847	p < 0.001	1.976	1.718 - 2.275	p < 0.001
N2	15.682	9.577 - 25.854	p < 0.001	5.483	3.132 - 9.673	p < 0.001
N3	0	NA	p = 0.964	0	NA	p = 0.962
Tumor size, cm						
< 2	Reference			Reference		
2 ~ 5	8.319	7.268 - 9.542	p < 0.001	1.864	1.581 - 2.202	p < 0.001
>= 5	16.269	13.825 - 19.172	p < 0.001	2.690	2.191 - 3.307	p < 0.001
Primary site						
Intestine	Reference			Reference		
Pancreas	2.289	2.046 - 2.560	p < 0.001	1.817	1.557 - 2.120	p < 0.001
Stomach	0.415	0.300 - 0.559	p < 0.001	0.656	0.454 - 0.927	p = 0.051
Histologic type						
Neuroendocrine tumor	Reference					
Neuroendocrine carcinoma	0.383	0.319 - 0.462	p < 0.001	1.033	0.831 - 1.290	p = 0.809
Other	1.820	1.521 - 2.188	p < 0.001	2.363	1.911 - 2.937	p < 0.001
Race						
Black	Reference			Reference		
Other	0.691	0.545 - 0.875	p = 0.010	0.894	0.678 - 1.178	p = 0.505
White	1.433	1.214 - 1.700	p < 0.001	1.232	1.016 - 1.500	p = 0.079
Sex						
Female	Reference					
Male	1.060	0.956 - 1.176	p = 0.355			
Age, years						
< 30	Reference			Reference		
30 ~ 60	3.761	2.417 - 6.245	p < 0.001	1.317	0.794 - 2.304	p = 0.393
>= 60	4.875	3.138 - 8.087	p < 0.001	1.202	0.725 - 2.102	p = 0.568

GEP-NETs, gastroenteropancreatic neuroendocrine tumors; HR, Hazard Ratio; CI, confidence interval; NA, Not Available.

### 3.3 Establishment and validation of a nomogram diagnostic model for distant metastasis in patients with GEP-NETs

**Figure 2:**
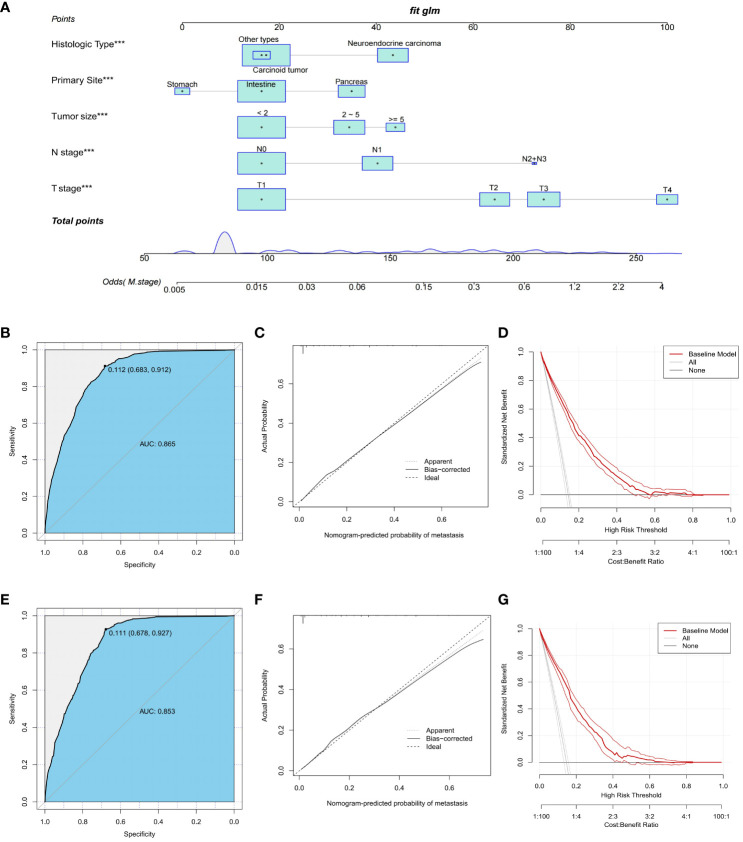
Construction and validation of a nomogram risk model for distant metastasis in GEP-NETs patients. **(A)** A nomogram risk prediction model for distant metastasis in GEP-NETs patients constructed on the basis of five independent risk factors (T stage, N stage, tumor size, primary site and histologic type). The model prediction performance was evaluated by ROC curves, calibration curves and DCA curves in the **(B-D)** training set and **(E-G)** validation set of the model group. AUC, area under curve. ***, p value<0.001.

**Figure 3:**
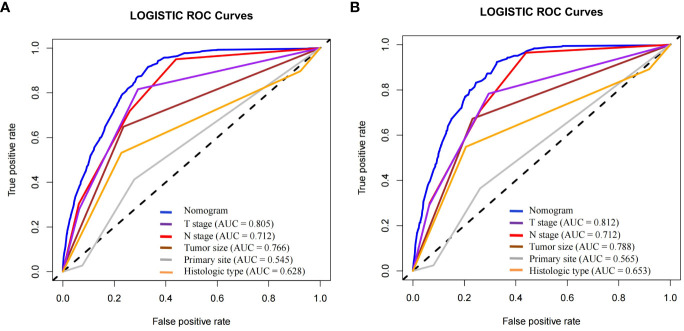
In the **(A)** training and **(B)** validation sets of the model group, the discrimination between the model and independent risk factors (T stage, N stage, tumor size, primary site and histologic type) for predicting distant metastasis in GEP-NETs patients was compared by ROC curves. AUC, area under curve; ROC, receiver operating characteristic.

To predict distant metastasis in GEP-NETs patients, we developed a nomogram risk prediction model based on five independent risk factors: T stage, N stage, tumor size, primary site and histologic type (**Figure 2A**). Next, we evaluated the ability of the model risk prediction by using several indicators. In the training set, the Area Under Curve (AUC) value of the ROC curve is 0.865 indicating that the model has a high degree of discrimination (**Figure 2B**). The calibration curve indicated that the model prediction curve had a high degree of agreement with the calibration curve indicating that the model had a high prediction accuracy (**Figure 2C**). The DCA curve demonstrated that the model had high clinical utility (**Figure 2D**). Internal validation was performed through the validation set. The results indicated that the model also possessed a high degree of discrimination (AUC = 0.853), accuracy and clinical utility in the validation set (**Figures 2E–G**). In addition, we also plotted ROC curves for the risk prediction of the five independent risk factors. The results indicated that the constructed nomogram model had a high discriminatory ability compared to individual risk factors, both on the training and validation sets (**Figures 3A, B**).

**Figure 4:**
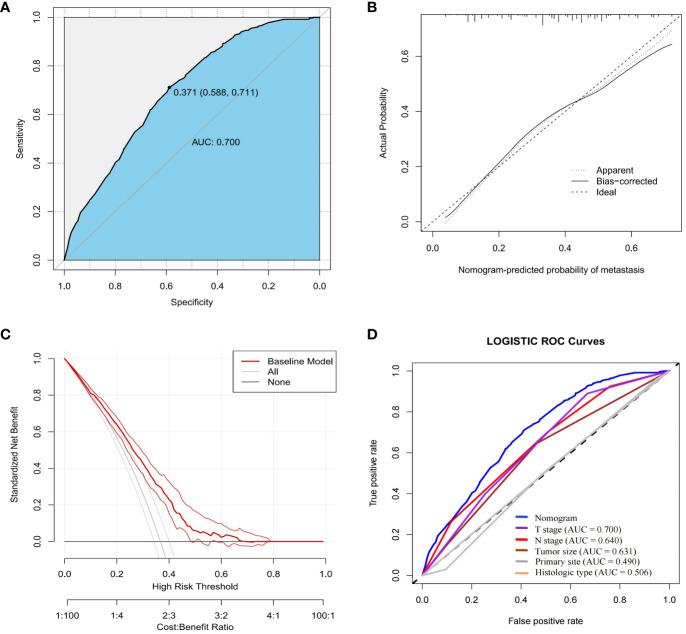
External dataset (validation group) to validate the nomogram risk model for distant metastases in GEP-NETs patients. The validation group assessed the model performance through **(A)** ROC curves, **(B)** calibration curves and **(C)** DCA curves. **(D)** Assessment of the differentiation between the model and the independent risk factors (T stage, N stage, tumor size, primary site and histologic type) through ROC curves. AUC, area under curve; ROC, receiver operating characteristic.

In order to further validate the ability of the model prediction, we selected a validation group of GEP-NETs patients from 2004-2009 for external validation. The results demonstrated that the nomogram model also presented good predictive ability in the validation group (AUC = 0.700; **Figures 4A–D**). Overall, the patient metastasis risk diagnostic model we constructed for GEP-NETs has good performance.

### 3.4 Analysis of prognostic factors for metastasis in GEP-NETs patients

In this study, a total of 1175 GEP-NETs patients in the model group had distant metastases (**Table 5**). Univariate Cox analysis revealed age, primary site, histologic type, T stage, N stage, primary site surgery, lymph node disposition and tumor size as prognostic-related factors in GEP-NETs patients (**Table 5**; p value < 0.05). Multivariate Cox analysis demonstrated that 30-60 years of age, neuroendocrine tumor histologic type, and tumor size >=5 centimeter were independent protective factors for the prognosis of GEP-NETs patients (HR < 1; p value < 0.05), whereas N2 staging and unoperated primary site were independent risk factors for the prognosis of GEP-NETs patients (**Table 5**; HR > 1; p value < 0.05).

**Table 5:** Univariate and multivariate Cox analysis of prognostic factors associated with distant metastases in GEP-NETs patients.

Characteristics	Total (n)	Univariate Cox analysis		Multivariate Cox analysis	
		HR (95% CI)	p value	HR (95% CI)	p value
Age	1175		< 0.001		
< 30	652	Reference		Reference	
30 ~ 60	510	0.524 (0.448 - 0.613)	< 0.001	0.491 (0.419 - 0.575)	< 0.001
>= 60	13	0.530 (0.237 - 1.186)	0.122	0.540 (0.241 - 1.210)	0.134
Sex	1175		0.112		
Male	606	Reference			
Female	569	0.887 (0.765 - 1.029)	0.113		
Race	1175		0.367		
White	957	Reference			
Black	123	1.152 (0.910 - 1.460)	0.240		
Other	95	1.140 (0.868 - 1.497)	0.345		
Primary site	1175		< 0.001		
Pancreas	436	Reference		Reference	
Intestine	708	0.516 (0.444 - 0.600)	< 0.001	1.131 (0.918 - 1.393)	0.249
Stomach	31	1.109 (0.720 - 1.708)	0.640	1.180 (0.758 - 1.836)	0.463
Histologic type	1175		< 0.001		
Other	123	Reference		Reference	
Neuroendocrine tumor	421	0.359 (0.279 - 0.461)	< 0.001	0.451 (0.341 - 0.596)	< 0.001
Neuroendocrine carcinoma	631	0.777 (0.621 - 0.973)	0.028	0.820 (0.638 - 1.055)	0.123
T stage	1175		0.011		
T4	355	Reference		Reference	
T3	487	0.937 (0.784 - 1.121)	0.479	1.041 (0.865 - 1.252)	0.670
T2	280	1.260 (1.035 - 1.534)	0.021	0.851 (0.686 - 1.055)	0.141
T1	53	0.834 (0.562 - 1.237)	0.367	0.760 (0.490 - 1.180)	0.222
N stage	1175		< 0.001		
N0	404	Reference		Reference	
N1	746	0.716 (0.615 - 0.835)	< 0.001	1.053 (0.884 - 1.254)	0.566
N2	25	2.249 (1.454 - 3.476)	< 0.001	2.267 (1.354 - 3.795)	0.002
Primary site surgery	1175		< 0.001		
Yes	766	Reference		Reference	
No	409	2.992 (2.573 - 3.478)	< 0.001	2.253 (1.616 - 3.142)	< 0.001
Lymph node dissection	1175		< 0.001		
Yes	692	Reference		Reference	
No	483	2.515 (2.165 - 2.920)	< 0.001	1.346 (0.979 - 1.850)	0.067
Tumor size	1175		< 0.001		
< 2	332	Reference		Reference	
2 ~ 5	615	0.586 (0.498 - 0.689)	< 0.001	0.843 (0.705 - 1.008)	0.061
>= 5	228	0.339 (0.267 - 0.430)	< 0.001	0.597 (0.451 - 0.789)	< 0.001

GEP-NETs, gastroenteropancreatic neuroendocrine tumors; HR, Hazard Ratio; CI, confidence interval.

### 3.5 Establishment and validation of a nomogram prognostic model for distant metastasis in GEP-NETs patients

**Figure 5:**
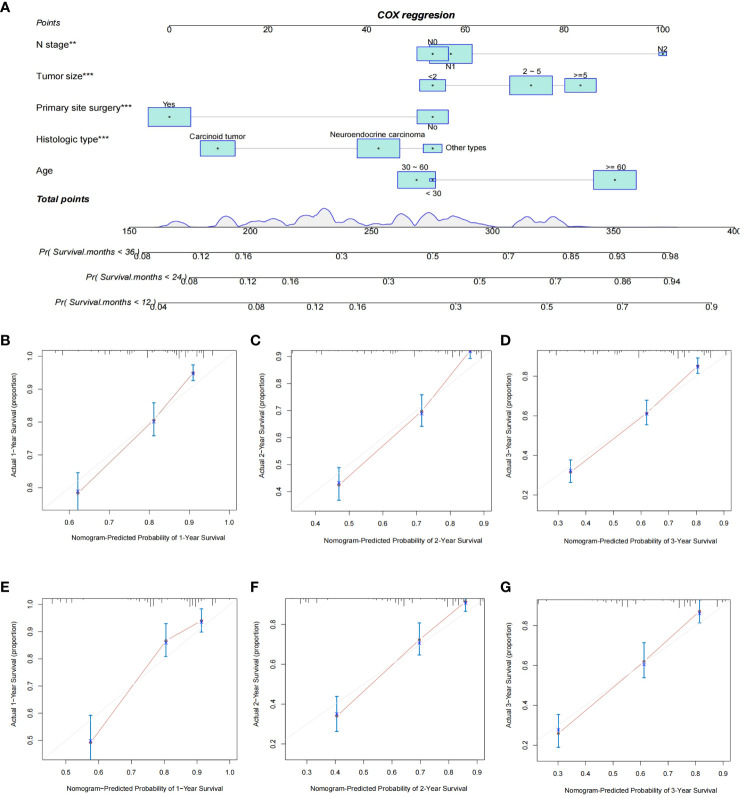
Construction and validation of a nomogram model for distant metastasis survival prediction in GEP-NET patients. **(A)** Construction of a nomogram survival prediction model for distant metastasis in patients with GEP-NETs based on independent prognostic factors (age, histologic type, tumor size, N stage, and primary site surgery). The accuracy of model survival predictions was assessed by plotting the model’s 1-,2- and 3-year prediction calibration curves in the **(B–D)** training and **(E–G)** validation sets of the model group. **, p value<0.01; ***, p value<0.001.

**Figure 6:**
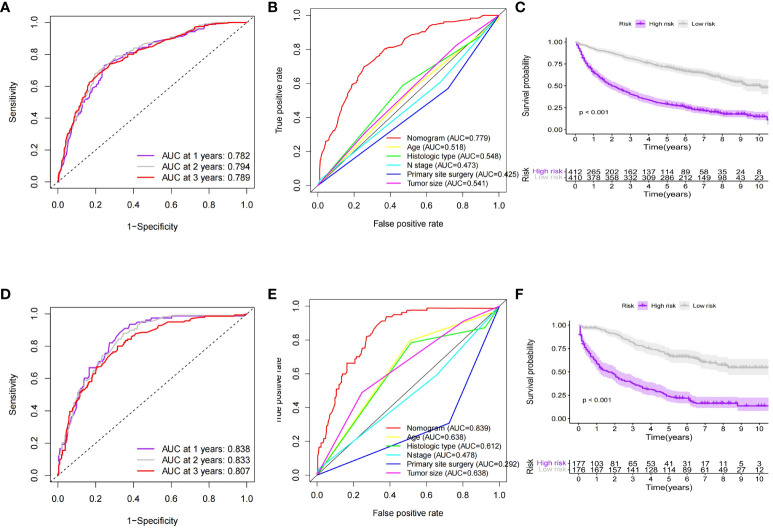
Evaluation of the predictive performance of the nomogram model for predicting survival of distant metastases in GEP-NETs patients. In the **(A)** training and **(D)** validation sets of the model group, ROC curves were used to assess the discrimination of 1-,2- and 3-year survival prediction in GEP-NETs patients with distant metastases. ROC curves assessed model and independent prognostic factors (age, histologic type, tumor size, N stage, and primary site surgery) for survival prediction differentiation in the **(B)** training and **(E)** validation sets of the model group. Comparison of differential survival of GEP-NETs patients of high- and low-risk groups in the model group according to median model survival prediction scores in the **(C)** training set and **(F)** validation sets of the model group (p value < 0.05). AUC, area under curve; ROC, receiver operating characteristic.

Based on independent prognostic factors in GEP-NETs patients, we constructed a nomogram survival prediction model to predict 1-, 2-, and 3-year survival in patients with distant metastases (**Figure 5A**). Next, we evaluated the performance of model survival prediction. The calibration curves indicated that the 1-, 2-, and 3-year survival prediction curves fluctuated slightly above and below the calibration curves in both the training and validation sets, indicating that our model has a high prediction accuracy (**Figures 5B–G**). The ROC curves demonstrated that the 1-, 2-, and 3-year survival predictions in the training set were well differentiated (**Figure 6A**), and the models in the validation set also exhibited good differentiation (**Figure 6D**). In addition, the ROC curves revealed that the model’s survival prediction differentiation was higher than the five independent prognostic factors in both the training and validation sets (**Figures 6B, E**). The patients in the test set and validation set were divided into high- and low-risk groups based on the median value of the model’s survival prediction score, and the results indicated that the survival of patients in the high-risk group was significantly lower than that in the low-risk group (**Figures 6C, F**; p value < 0.05). This is provided further evidence that the model we constructed has good survival differentiation ability.

**Figure 7:**
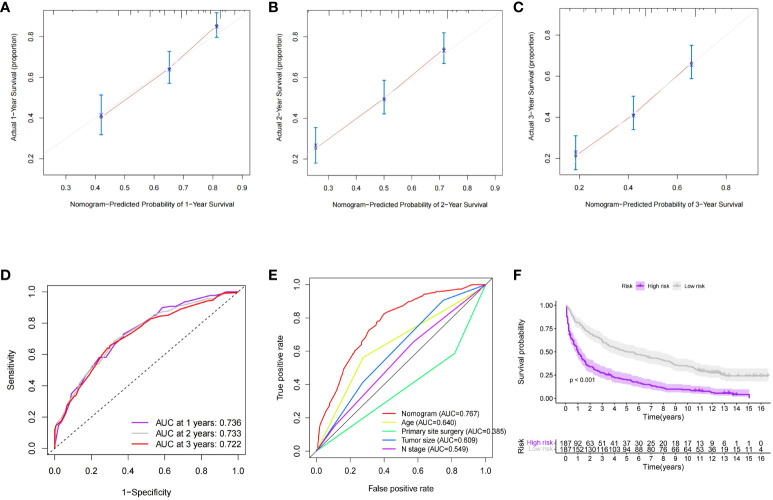
External dataset (validation group) to validate the nomogran survival prediction model for distant metastases in GEP-NETs patients **(A–C)** The calibration curves assessed the accuracy of model 1-2-and 3-year sunival predictions in the validation group. In the validation group, **(D)** ROC curves were used to assess the discrimination of model 1-2-and 3-yeur survival predichom, **(E)** and to compare model discrimination with independent prognostic factors (age, histologic type, tuanor size, N stage, and primary site surgery) **(F)** In the validation group, the differential survival in the high and low-risk groups was compared according to the median value of the model's survival prediction score (p value <0.05) AUC, arca under curve.

In order to further validate the survival prediction ability of the model, we selected patients with distant metastases of GEP-NETs from 2004-2009 as the validation group for external validation. Predictive performance assessment revealed that the 1-,2- and 3-year survival prediction curves of patients in the validation group had a high degree of agreement with the calibration curves (**Figures 7A–C**), and the ROC curves also demonstrated a high degree of model discrimination (**Figures 7D, E**). In addition, the patients in the validation group could be well divided into high- and low-risk groups based on the median value of the model’s predictive scores, and the prognosis of patients in the high-risk group was significantly worse than that of the low-risk group (**Figure 7F**; p value < 0.05). In summary, the nomogram survival prediction model we constructed has excellent performance in predicting prognosis of GEP-NETs patients with distant metastases.

## 4 Discussion

The incidence of GEP-NETs is increasing every year and has become a serious threat to human health (10, 17). Surgery is the primary modality for patients with early-stage GEP-NETs and has helped to greatly improve long-term survival, but once metastasis develops patients have a poorer outcome (17–19). Currently, the treatment of patients with advanced GEP-NETs faces many problems. The precision therapeutic methods of molecular targeting have achieved remarkable results in the field of oncology, but the therapeutic application in GEP-NENs is still immature, and some targets are controversial (20). Peptide receptor radionuclide therapy-based combination therapies with and anti-vascular endothelial growth factor drugs with standard chemotherapy have achieved good results, but still need to be studied in larger trials (21). In addition, evidence for antiproliferative therapies with growth hormone analogs such as octreotide and lanreotide is increasing, but some clinical indications remain controversial (22). Therefore, it is significant to analyze the risk factors of distant metastasis in GEP-NETs patients and formulate effective preventive measures so as to improve the prognosis of patients. This is also consistent with the modern medical concept of precision treatment of tumors (23, 24).

The first applications of nomograms in medicine originated in the 19th century (25, 26). Currently, probabilistic nomograms are most commonly used to determine the probability of an individual’s specific events, which is determined by multivariate dichotomous regression-based or Cox proportional risk models (27). Because the nomogram has the advantages of simplicity, accuracy, and incorporation of disease characteristics, it is now widely used in clinical research and clinical decision-making (27). Study demonstrated that nomograms exhibited excellent predictive performance in the assessment of survival prediction in small cell lung cancer, hepatocellular carcinoma and glioma (28–30). The nomograms are also applicable to the prediction of stomach, breast and thyroid cancers metastasis risk (31–33). Based on technologies such as imaging pictures and pathology slides, nomograms can predict tumor biology and treatment outcomes (34–36). In addition, nomograms can predict postoperative complications in hepatocellular carcinoma patients based on liver stiffness (37). Therefore, nomograms have important clinical applications.

Broadbent et al. demonstrated that tumor size, tumor invasiveness, surgical resection of the lesion, and lymph node metastasis were significantly related to patient prognosis in patients with GEP-NETs (38). In this study, we included these clinical factors through the SEER database. Risk factors and prognostic factors for distant metastasis in GEP-NETs patients were analyzed, and nomograms were constructed to predict the risk of distant metastasis and prognostic predictions for patients. The nomogram models we constructed exhibit excellent prediction performance, both internally through the validation set divided by the model group and externally through the validation group. Therefore, our newly developed nomogram models can be effective in predicting the risk of distant metastasis in GEP-NETs patients and evaluating the prognosis of the patients, so as to adopt targeted clinical prevention or treatment programs. Specifically, for patients diagnosed with GEP-NETs, we collected clinical parameters, predicted the risk of distant metastasis by the risk of distant metastasis model and predicted the prognosis of patients by the prognostic model. Based on the risk of metastasis assessed by the model and the prognosis predicted by the model, we develop a clinical treatment plan to prevent distant metastasis and thus improve the survival of the patients. Therefore, the nomogram models we constructed have great clinical significance.

Currently, there are several articles reporting studies on constructing models of GEP-NETs, which also suggests that research on the model of GEP-NETs is a hot research direction in the current clinic. Adrienne B Shannon et al. screened 12,228 patients with stage I-III nonfunctional GEP-NETs who underwent surgical resection and lymph node clearance through the National Cancer Database to establish a nomogram prediction model for lymph node metastasis (39). Cheng Fang et al. screened 10,236 GEP-NETs patients with clinical information from the SEER database and constructed a nomogram model to predict 3- and 5-year survival (40). Compared with these models, the advantage of the model we constructed is that the study is more comprehensive. The included clinical factors related to GEP-NETs constructed a distant metastasis prediction model and a distant metastasis prognosis model, which systematically studied the risk and prognosis of patients with distant metastasis of GEP-NETs.

However, there are some shortcomings in this study. First, although we adopted an internal validation combined with external validation to demonstrate the accuracy of the nomogram models we constructed, these data were derived from publicly available databases and still lack further validation from our own clinical follow-up data. To address this problem, we plan to collect more comprehensive clinical follow-up data in future studies to strengthen the validation and accuracy of the model. Second, we selected patients with GEP-NETs from 2004-2009 in the SEER database as the validation group. Due to the long period of time, some of the data, such as the histologic type, is discrepant from the most recent data, which affects the performance of our model in external validation. To remedy this shortcoming, future studies will consider the use of updated datasets with a comprehensive analysis of new clinical factors to enhance the timeliness and applicability of the model. Finally, due to the limitations of the SEER database, important clinical information such as pathologic grading, chemotherapy, and radiation therapy are missing for GEP-NETs patients (41–43). These clinical factors have a significant impact on the prognosis of patients with GEP-NETs, however, this important information was missing from the model we constructed. Therefore, we intend to incorporate these clinical factors in subsequent studies to enhance the comprehensiveness and usefulness of the model. Meanwhile, we also plan to explore more new factors related to prognosis, such as molecular biomarkers and gene expression characteristics, to further enhance the predictive ability of the model.

In conclusion, we constructed a new distant metastasis risk nomogram model and a new prognostic nomogram model for GEP-NETs patients, which provides a decision-making reference for individualized treatment of clinical patients. Although the models we constructed have high predictive performance, however, they still face many problems for clinical applications. Future research on the modeling of GEP-NETs should focus on translating to practical clinical applications.

## Data availability statement

The original contributions presented in the study are included in the article/**Supplementary Material**. Further inquiries can be directed to the corresponding author.

## Ethics statement

The studies involving humans were approved by the Ethics Committee of Changzhou Second People’s Hospital affiliated with Nanjing Medical University. The studies were conducted in accordance with the local legislation and institutional requirements. The participants provided their written informed consent to participate in this study.

## Author contributions

XL: Conceptualization, Writing – original draft. YF: Data curation, Visualization, Writing – review & editing. JT: Data curation, Visualization, Writing – review & editing. ML: Supervision, Writing – review & editing.
